# Minimal Excision and Primary Suture is a Cost-Efficient Definitive Treatment for Pilonidal Disease with Low Morbidity: A Population-Based Interventional and a Cross-Sectional Cohort Study

**DOI:** 10.1007/s00268-016-3828-z

**Published:** 2016-11-30

**Authors:** Kaveh Khodakaram, Joachim Stark, Ida Höglund, Roland E. Andersson

**Affiliations:** 1 Department of Surgery, Sahlgrenska University Hospital, Gothenburg, Sweden; 2 Department of Surgery, County Hospital Ryhov, 551 85 Jönköping, Sweden; 3 Department of Surgery, Värnamo Sjukhus, Värnamo, Sweden; 4 Department of Clinical and Experimental Medicine, Linköping University, Linköping, Sweden

## Abstract

**Background:**

Conventional treatment of pilonidal disease with wide excision is associated with high morbidity. We describe the short- and long-term results and the impact on the health care system of a simple operation performed in the office under local anaesthesia, consisting of minimal excision of pilonidal sinuses with primary suture—the modified Lord–Millar operation (mLM).

**Methods:**

All patients operated with mLM from February 2008 till November 2012 were prospectively followed for recurrence by telephone interviews and examination of symptomatic patients till July 2015. The outcome is compared with that in all patients operated with conventional wide excision from January 2003 till February 2008. The effects on the health care system of a consistent use of mLM is analysed by comparing the management of all patients with pilonidal disease at three hospitals during 2013 and 2014.

**Results:**

Some 129 patients underwent conventional surgical treatment, and 113 had the mLM operation. The mLM operation was more often performed under local anaesthesia, was less often admitted to hospital, had fewer post-operative health care visits (2.4 vs. 14.6, *p* < 0.001) and a shorter sick leave (1.0 vs. 34.7 days, *p* < 0.001) indicating faster wound healing. The estimated 5-year recurrence rate was similar (32 vs. 23%, *p* = 0.091). The cost per operated patient was lower (2231 vs. 6222 EUR, *p* < 0.001). The hospital consistently applying the mLM operation used less resources for pilonidal diseased patients (34,545 vs. 77,421 EUR per 100,000 inhabitants and year).

**Conclusions:**

The mLM operation is simple, cost-efficient and has low morbidity and good long-term results.

## Introduction

The aetiology of pilonidal disease is not completely understood. A foreign body reaction to subcutaneous accumulation of hairs with recurrent abscesses and eventually the development of chronic discharging tract is usually involved, but hairs are not always found. The origin or entrance for these hairs may be a barely visible pit or larger epithelial lined sinus openings in the midline [1]. Intermittent or continuous discharge is common from the midline sinuses or from the tract that drains the abscess, often located proximal and lateral to the midline sinuses [2]. See fact box for further information on definitions.

Several surgical methods are described, and the optimal method is controversial [3]. Many studies are flawed because of defects in study design, unclear patient selection and exclusions not stated, short or incomplete follow-up, and failure to assess treatment costs and patient inconvenience [4]. Traditional treatment involves a large excision under general anaesthesia, often with an overnight stay in hospital. The wound is left open for secondary healing or closed with or without surgical flaps. Wound complications, needing frequent dressings and sick leave, are common [4]. Studies have shown 20% unhealed wounds at 3 months and up to 22% recurrence rate after 5-year follow-up [5].

Less extensive methods are described but have not gained widespread acceptance [6, 7]. In 1965 PH Lord and DM Millar described a method with removal of each pilonidal pit or sinus through separate excisions with minimal margin and cleaning of the cavity from hair and granulation tissue, leaving the small wounds open for secondary healing [8]. Results of this method have been reported by others and have been popularised by Gips et al. who uses trephines for the excision [9–11].

In February 2008 we introduced a modification of the Lord–Millar operation (mLM). Unlike the original method we closed the wound after the excision. The rational is that this will stop further contamination and will hasten the healing of the wound. To secure the safety of this modification we registered prospectively all operated patients. We hypothesise that the mLM, is safe, will give lower morbidity and similar long-term outcome as the conventional wide excision. After the introduction the method has been consistently used at our hospital for all cases operated for pilonidal disease. We analyse the impact of this management on the health care system by comparing the management of all patients with pilonidal disease at the three hospitals, each serving a defined geographic district, in Jönköping county in 2013 and 2014. We hypothesise that the consistent use of the mLM is cheaper for the health care system and does not lead to accumulation of recurrences.

## Materials and methods

Jönköping county, population 336,866 inhabitants in 2010, has three health care districts [Jönköping (A), Höglandet (B) and Värnamo (C) health districts]. Each district is served by one hospital, some healthcare centres and a few private health care providers. All patient contacts for these providers, including contact with nurses, are registered in the county’s electronic administrative database with an ICD10 code for the diagnosis and an eventual intervention. From this database we identified all health care contacts with an ICD10 code L05* from January 2003 till December 2014. Patients treated surgically were identified by the intervention code QBG20 or QBE10. Surgical treatment for pilonidal disease is only performed at the three hospitals.

### Pre-intervention cohort using conventional wide excision

Information about patients with a primary operation for pilonidal disease at hospital A from January 2003 till February 2008 was obtained from the administrative database and a review of the patients’ files. Information on surgical method, choice of anaesthetics, number of visits pre- and post-operatively, length of stay, sick leave after the primary intervention, date of eventual recurrence and of surgical treatment for recurrence was collected. The patient files has high quality as most of them are included in a previously published prospective randomised trial of placing a gentamicin–collagen sponge in the wound to reduce the rate of wound infection and recurrences [12].

The traditional treatment at hospital A was wide excision of the sinus, the cavity and all tracts through a wide symmetric excision with diathermy under general anaesthesia. Methylene blue was used to search for tracts. The wound was closed in one layer in the midline. Prophylactic antibiotic treatment was not given. Some surgeons used a more limited excision of the cavity and lateral tract sparing as much skin as possible. Two patients were operated with the Karydakis method [13]. Most patients were admitted for one night stay in hospital. Recurrences after the wide excisions were treated with repeat wide excision and healing by secondary intention or flap techniques.

We made a long-term follow-up of all patients from this period by a postal questionnaire in August 2011. Those not responding to the questionnaire were contacted by telephone. Patients with symptoms suggesting recurrence were invited for re-examination and reoperation if needed. Patients were considered to have recurrent disease if they had required reoperation or reported symptoms of local pain, intermittent swelling or purulent discharge at the follow-up.

### Intervention cohort using the Modified Lord–Millar method

All patients with a primary operation for pilonidal disease with the mLM operation at hospital A were prospectively registered from the introduction in February 2008 till November 2012. The registered information includes extent of disease, type of intervention, choice of anaesthetics, length of stay in hospital, sick leave and number of visits pre- and post-operatively. A few patients that were operated with wide excision during a transition period after the introduction of mLM are included in the pre-intervention cohort.

**Fig. 1:**
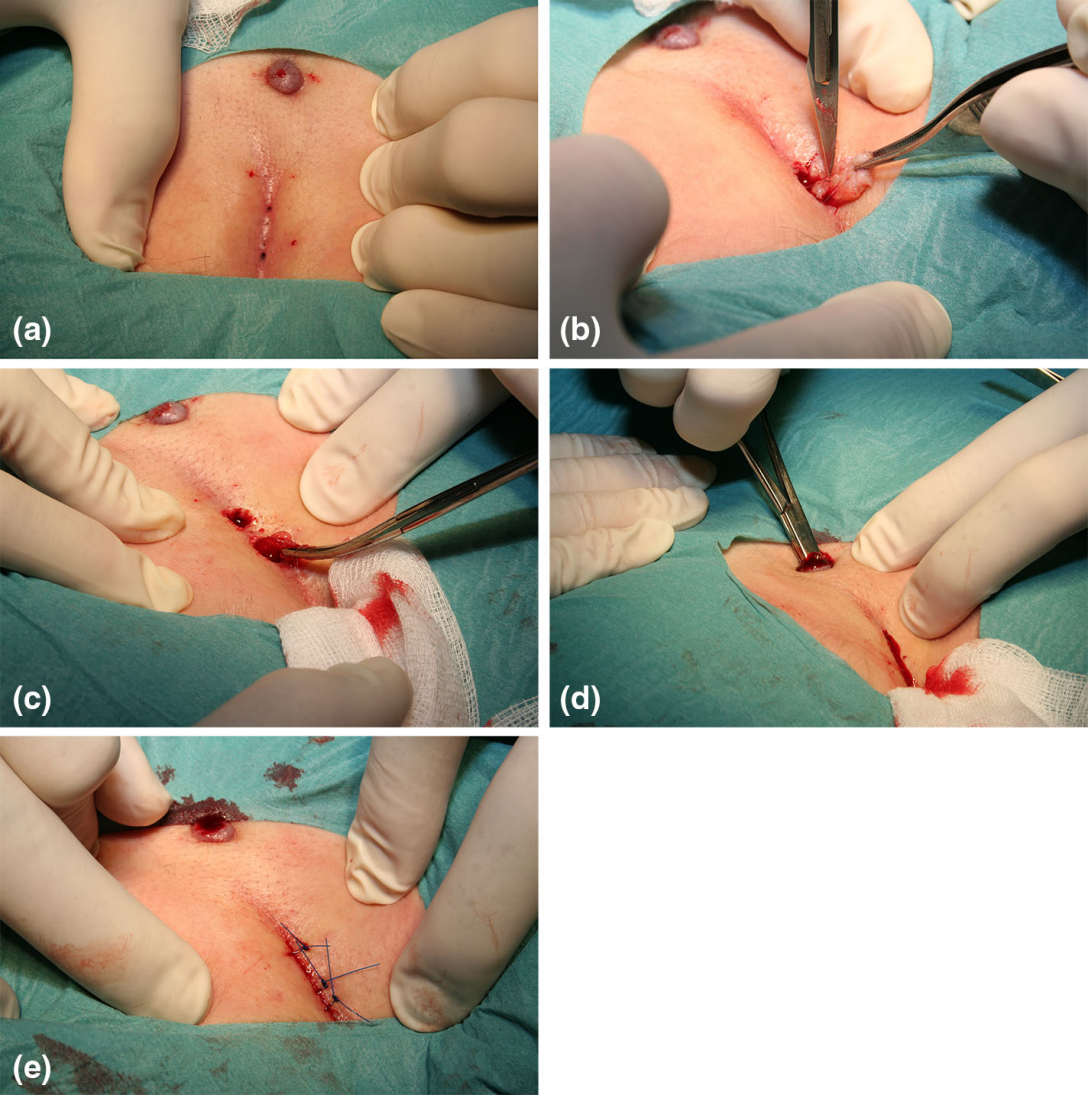
**a** Pilonidal sinuses and a proximal lateral tract. The area is infiltrated with local anaesthetic. **b** Pilonidal sinuses in the midline are excised through separate incisions with minimal margin. **c** All hairs and possible granulation tissue is removed with hemostat or a surgical spoon. **d** The tract is also searched and cleansed from hairs, using a hemostat. **e** In contrast to the original method the wound is closed primarily. The lateral tract is left open for drainage

The surgery is done under local anaesthesia in a 20-min outpatient visit at the office. The patient is placed in prone position. The skin is shaved and cleansed with chlorhexidine solution. A mixture of Carbocain 1% (14 ml) and sodium bicarbonate 5% (6 ml) is used for local anaesthesia. All pits and sinuses are excised down to the subcutaneous fat through separate excisions with a minimal margin with scalpel (Fig. 1). The amount of tissue excised at each place is <1 cm^3^. Hair and granulation tissue is meticulously removed from the cavity and the lateral tract through the excisions and the tract opening using a haemostat, a surgical spoon or a strip of gauze. A lateral tract that is closed is opened by the haemostat or a scalpel and cleansed. In contrast to the original method the wounds after the excisions are sutured with closely spaced non-absorbable sutures. The tract is left open for drainage. Patient is encouraged to go to school or work the same day. No sick leave and no antibiotics are prescribed. Sutures are removed after 10 days. If the wound is not healed at the removal of sutures the patient may come back weekly for change of dressings and shaving. Recurrences after mLM have the same appearance as at the primary operation and are treated with the same small excisions. This is repeated if needed.

All patients were followed up through a telephone interview after 1, 3 and 12 months. A long-term follow-up was done in July 2015 with a review of the patients’ files, a telephone interview and examination of patients with symptoms suggesting a recurrence. Reoperations for recurrences, the surgical method used and the outcome were noted.

### Comparing management and results at three hospitals

Since the introduction in 2008, the modified Lord–Millar method has become the only method used for the primary treatment of pilonidal disease and for recurrences after mLM at hospital A irrespective of the seriousness and extent of disease. At hospital B the modified Lord–Millar method is partly used since 2013 for primary operations, but wide excision are used for recurrences. Hospital C uses traditional methods.

The effects of the change in method on the health care system were analysed by comparing the management and outcome for all patients, also non-operated, with a diagnosis of pilonidal disease in the county’s three districts during 2013 and 2014. Information about each health care contacts for pilonidal disease—incision of abscess, surgical treatment, surgical method used, if the indication for surgery was primary or recurrent disease, choice of anaesthetics and locale where the operation was performed and pre- and post-operative contacts with nurses for wound care—was obtained from the administrative database and a review of the patients files. For patients with a primary intervention the number of visits pre- and post-operatively, length of stay and sick leave were analysed. Based on the operative report we classified the type of operations as the mLM operation, excision leaving the wound open, excision plus primary suture or excision with a Limberg flap [14].

### Cost analysis

The county’s health care providers are reimbursed for each patient contact, as recorded in the administrative database, according to the price list in Table 1. We used these prices and the recorded contacts to estimate the costs for the care of the patients with pilonidal disease for the pre- and post-intervention period and for the three health care districts in the cross-sectional analysis. We have not included the patients charge or the sick leave compensation in the calculations.

**Table 1:** Reimbursement, in Euro (EUR), assigned to items according to the pricelist set by Jönköping county’s administration

Item	Reimbursement in EUR
Doctors visit, hospital	574.24
Doctors visit, care centre	168.29
Nurse visit, hospital	229.69
Nurse visit, care centre	67.34
Operation at surgeons office in local anaesthesia	574.24
Operation at theatre in general anaesthesia (30 min)	821.85
Recovery room after general anaesthesia (1 h)	145.81
Ward costs (1 day)	1309.98

### Statistical methods

Differences were analysed with *t* test, Mann–Whitney *U* test, Kruskal–Wallis test, the Chi-squared test and Fishers exact test, where applicable. Recurrence-free survival was analysed using the Kaplan–Meier method and the log-rank test. Risk factors for recurrence were analysed using Cox regression. The study was approved by the regional ethics committee, in Linköping University Hospital.

## Results

### Pre- and post-intervention cohorts

Some 129 patients underwent conventional extensive surgery at hospital A in the pre-intervention period and 113 patients with the mLM method after the introduction in February 2008. During a transition period during 2008 and 2009 some surgeons continued using the more extensive methods in 21 cases. These are included in the pre-intervention cohort. Since 2009 all primary operations for pilonidal disease has been done using the mLM method.

The intervention cohort includes more women, more patients with a history of abscess and fewer with a lateral tract (Table 2). They were more commonly operated under local anaesthesia, had fewer admission to hospital and a shorter length of stay. They had fewer visits both pre- and post-operatively and shorter sick leave, indicating faster wound healing. The reimbursement for the health care system is lower for patients operated with mLM.

**Table 2:** Demography, type of intervention and outcomes of the patients with primary surgical treatment for pilonidal disease at hospital A in 2003 till November 2012

Variable	Surgical method		
	Conventional wide excision	Modified Lord–Millar	*p* value
Total numbers	129	113	
Male sex, number (%)	116 (90)	86 (76)	0.004
Age, years, mean (SD)	28.5 (10.7)	27.4 (8.4)	0.41
Number of visits, mean (SD)			
Pre-operatively	2.6 (3.2)	1.6 (1.9)	0.006
Post-operatively	14.6 (28.2)	2.4 (3.6)	<0.001
History of abscess, number (%)	72 (56)	89 (79)	<0.001
Lateral tract, number (%)	95 (74)	64 (57)	0.019
Mean length of stay, nights (SD)	0.9 (0.6)	0.04 (0.2)	<0.001
Local anaesthesia, number (%)	9 (7)	108 (96)	<0.001
Sick leave, days, mean (SD)	34.7 (64.7)	1.0 (3.5)	<0.001
Follow-up, years, mean (SD)	5.1 (2.6)	3.3 (2.1)	<0.001
Estimated recurrence at 5 years, % (95% CI)	23 (16–31)	32 (23–43)	0.091
Reimbursement per patient, EUR, mean (SD)	6222 (6802)	2231 (1109)	<0.001

### Recurrences

**Fig. 2:**
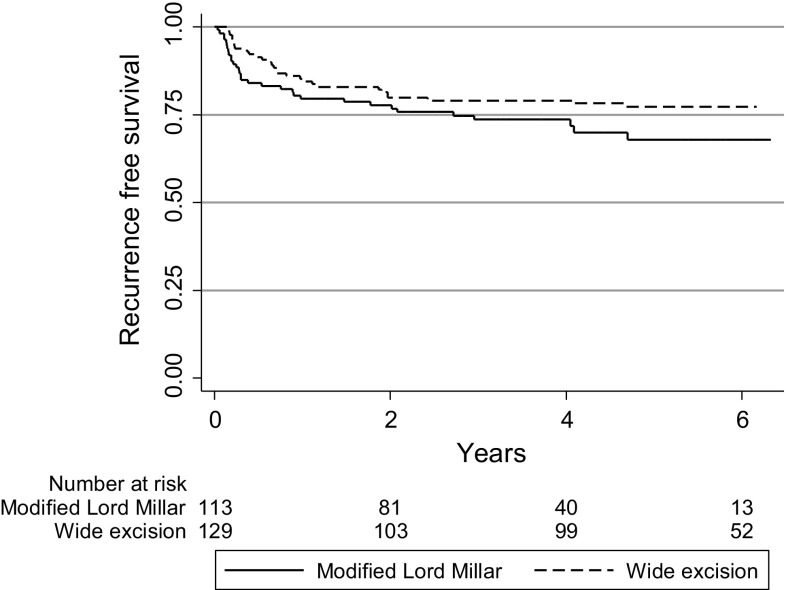
Kaplan–Meier curve for the recurrence-free survival after primary operation for pilonidal disease comparing the patients operated with wide excision and with the modified Lord–Millar method

After a mean follow-up of 3.3 versus 5.1 years the estimated 5-year recurrence rate was similar after mLM compared with the pre-intervention cohort (32 vs. 23%, log-rank test *p* = 0.091) (Fig. 2). Age, sex, history of abscess or presence of lateral tract was not associated with risk of recurrence after mLM (data not shown).

**Fig. 3:**
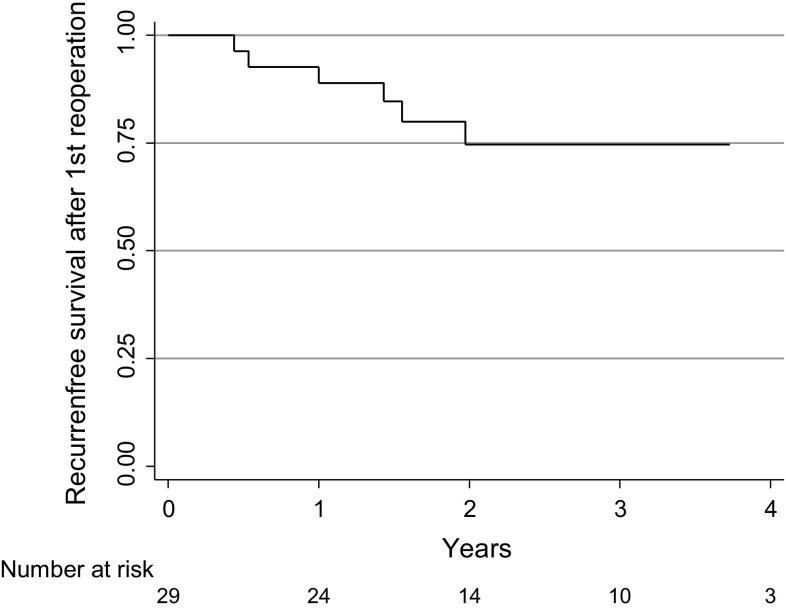
Kaplan–Meier curve for the recurrence-free survival for the patients operated for recurrence after operation with the modified Lord–Millar method, using the same method again

The estimated recurrence rate in the 29 patients that were re-operated with a second mLM operation was 25% (95% CI 12–49) at 3 years follow-up (Fig. 3). At the long-term follow-up in July 2015 six of the 113 patients (5%) had not healed, four with a first, one with a second and one with a third recurrence. Three patients complaining of symptoms at the telephone contact did not come for the examination. 21 patients were lost to the long-term follow-up.

### The impact on health care system

Some 243 patients had 814 health care contacts for pilonidal disease in the county during 2013 and 2014, giving an incidence rate of 36 per 100,000 inhabitants/year. The proportion treated with surgery and the proportion of operations for recurrences did not differ between the districts. The district served by hospital A, which used almost exclusively the mLM method, had the lowest rates of both pre- and post-operative health care contacts, lowest utilisation of hospital resources and prescribed fewer days of sick leave and had the lowest costs for this group of patients with pilonidal disease (Table 3).

**Table 3:** Comparison of the management of patients diagnosed with pilonidal disease in three districts of Jönköping county, each serving a defined population, in 2013 and 2014

	Health care district in Jönköping county			*p* value
	*A*	*B*	*C*	
Population	145,000	110,000	85,000	
Number of patients with pilonidal disease	133	72	62	
Number of patients with pilonidal disease per 100,000 inhabitants/year	46	33	36	0.051
Number of health care contacts	211	267	336	
Number of health care contacts per 100,000 inhabitants/year	73	121	198	<0.001
Age, mean (range)	27.2 (13–58)	27.6 (14–61)	29.9 (15–65)	0.33
Male sex (%)	70	79	66	0.21
Number of operated patients (%)	84 (63)	42 (58)	31 (50)	0.22
Number of operations^a^	103	55	37	
Number of operations for recurrences (%)^b^	29 (28)	15 (27)	12 (32)	0.85
Surgical method used				
Excision + open	0	7	10	
Excision + suture	2	10	20	
Limberg flap	0	0	7	
Modified Lord–Millar	101	38	0	<0.001
Operation in the office (%)	102 (99%)	40 (73%)	4 (11%)	<0.001
Operation in local anaesthesia (%)	103 (100%)	45 (82%)	5 (14%)	<0.001
Number of health care contacts, mean (range)^c^				
Pre-operatively	4.6 (1–29)	13.3 (1–153)	8.5 (1–49)	0.041
Post-operatively	3.0 (1–24)	13.2 (1–151)	17.1 (1–122)	<0.001
Prescribed sick leave, days, mean (range)^c^	0 (0–0)	2.3 (0–28)	15.8 (0–116)	<0.001
Reimbursement/patient with pilonidal disease, mean (SD)^d^	753 (915)	1146 (1051)	2123 (2367)	<0.001
Reimbursement/100,000 inhabitants/year^d^	34,545	37,520	77,421	<0.001

^a^Patients may have multiple operations

^b^Includes all operations for recurrences, including patients with a primary operation at other hospitals or in another period

^c^Results for patients with primary operation for pilonidal disease

^d^Reimbursement calculated for the management of all patients with pilonidal disease including non-operated

## Discussion

This study shows that the consistent use of the mLM operation for treating pilonidal disease is cost-efficient with low post-operative morbidity and long-term results comparable with more extensive methods, both compared with historical controls and in comparison of the treatment of pilonidal disease between three hospitals. The method also consumes less resources for the health care system and the society. The operation is performed as an outpatient procedure at the office under local anaesthesia with no need for hospital admission or sick leave. Most patients can return to work or school the same day.

In the original method the wounds after the excisions were left open for healing by secondary intention, in accordance with traditional surgical principles not to close an infected cavity. The disadvantage is prolonged time to healing. It also leaves an entrance for contagious material and hairs with risk for recurrent sinus. Bascom, in his similar pit-picking procedure [15], closed the wounds after the excision of the sinuses in the midline but added a lateral incision for excision of inflamed tissue and drainage, which was left open. This wound may take up to several months to heal [16].

Our modification consists of suturing the wounds. This will shorten the healing time, stop further contamination and prevent the formation of new sinuses. Although we did not prescribe any antibiotics we did not observe any adverse effects. There was no early abscess formation. Most wounds were healed when the sutures were removed after 10 days, as shown by the few number of post-operative visits.

Our estimated recurrence rate of 32% may seem high but is similar to many others using more extensive methods, although the reported recurrence rate after surgical treatment for pilonidal cyst varies considerably between studies [4]. Possible explanations are differences in definitions and method and length of follow as recurrences may come after many years [17, 18]. We have made an effort to reach all our patients for follow-up, and all patients having symptoms suggesting recurrence were clinically assessed.

However, recurrence rate is an inadequate outcome that does not reflect the degree of impairment in the quality of life for the patients. The recurrences after the mLM procedure have the same appearance as before the operation and is often less troublesome. At reoperation for recurrence the same surgical method can be applied with the same success rate, reaching a long term healing rate >90% after two operations.

The differences between the patients in the pre-and the post-intervention periods may suggest a difference in the selection of patients for surgery. However, the study is population-based, i.e. all patients having treatment for pilonidal disease from the hospitals catchment population during the study period are included with no selection. This allows a comparison between the pre- and post-intervention period as well as the comparison between the health districts. mLM has completely replaced the traditional surgical methods at hospital A. It is used for all cases irrespective of the seriousness of disease and consistently also for recurrences after mLM. The results from 2013 to 2014 show no increase in operations for recurrences. The comparison between the three health districts supports that the good results of mLM is not due to selection, is lasting and does not create a pile up or recurrences.

The minimal invasive method of the mLM is a simple, cost-efficient surgical method to treat pilonidal sinus disease with low frequency of wound complications. The same method can be used for recurrences providing good long-term results.

## Figures and Tables

**Fig. 4:**
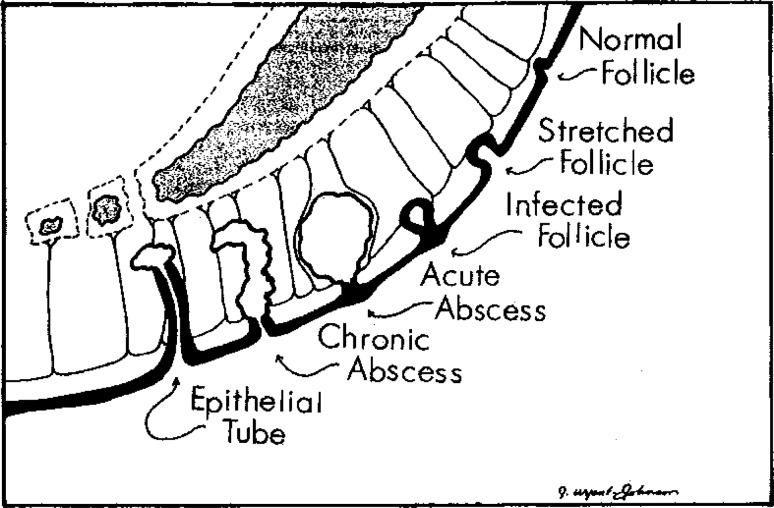
The stages of pilonidal disease according to Bascom

**Fig. 5:**
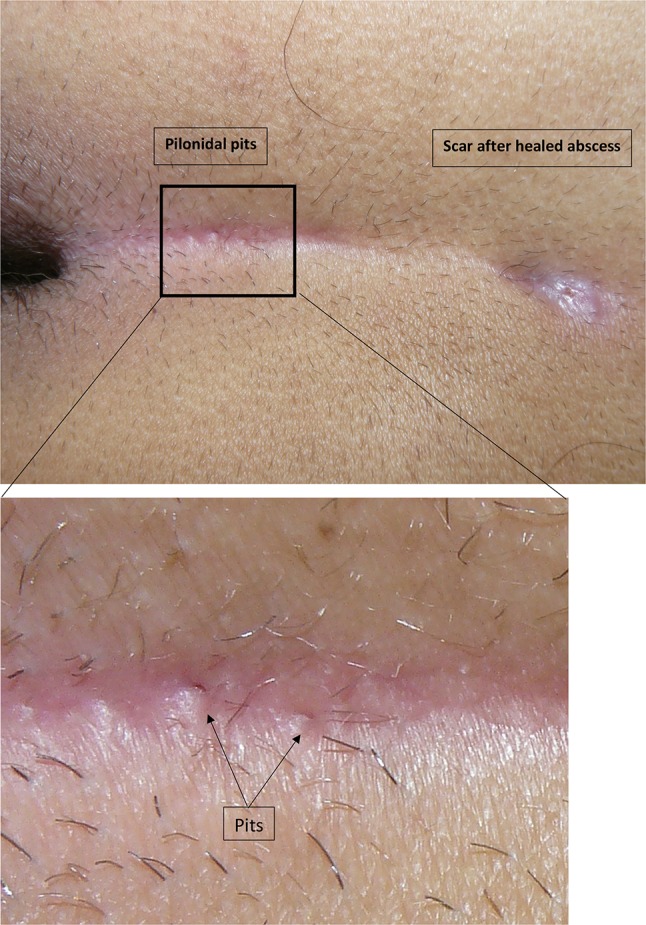
An early stage of pilonidal disease, showing a cranial scar after recurrent and healed abscess, and a two distal discrete pits. These pits are the entry for the infection

**Fig. 6:**
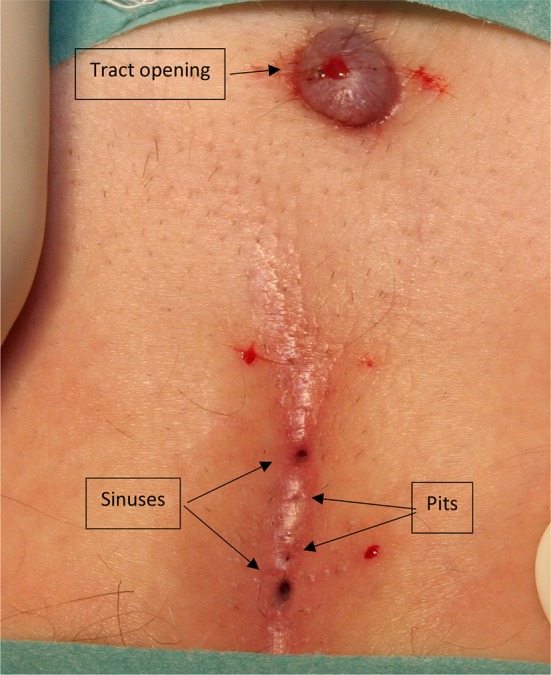
Fully developed disease with pits, sinuses and lateral tract

**Fig. 7:**
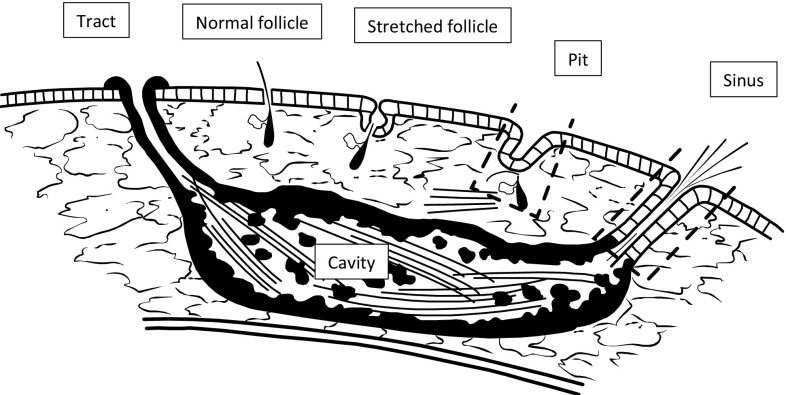
Sagittal view of pilonidal disease. The pit is the result of a deformed hair follicle. Often with subcutaneous accumulation of hairs. The sinus is an epithelial tube after destruction of the follicle, with connection to the cavity, which contains granulation tissue and often hairs. The cavity drains through the tract. The dashed lines indicate the minimal excisions needed. (Modified from an original drawing by Gips et al. [11])

## Fact box

Bascom has proposed a scheme for the development of the stages of the pilonidal disease (Fig. 4) [15]. According to this scheme the first stage is a “stretched follicle”. The early stage is also called “pilonidal pit” (Fig. 5). This can be seen as a discrete retraction. Under such a retraction an accumulation of hairs can be found. In a later stage an epithelial tube develops, the “pilonidal sinus”, which has contact with a cavity, often filled with granulation tissue and sometimes also hair (Figs. 6, 7). This cavity is the result of repeated abscess and can eventually result in chronique discharge from a tract, often located proximal and lateral to the sinus and the cavity.
